# Supplementary material for: Cost-effectiveness of trastuzumab deruxtecan for previously treated HER2-low advanced breast cancer
Source: PLoS One. 2023 Aug 24;18(8):e0290507. doi: 10.1371/journal.pone.0290507 (PMC10449172; doi:10.1371/journal.pone.0290507)

S1 Fig. Model Fitting Analysis.

To obtain the best model fit, the following investigations were carried out using trastuzumab deruxtecan or chemotherapy as the model fit baseline, respectively. Based on AIC and BIC (Supplementary Table 1). CPS, combined positive score.

(A) Model-fitted versus original K-M curves for trastuzumab deruxtecan in HER2-Low advanced BC patients, log-logistic and lognormal models were used to fit the OS and PFS K-M of trastuzumab deruxtecan.

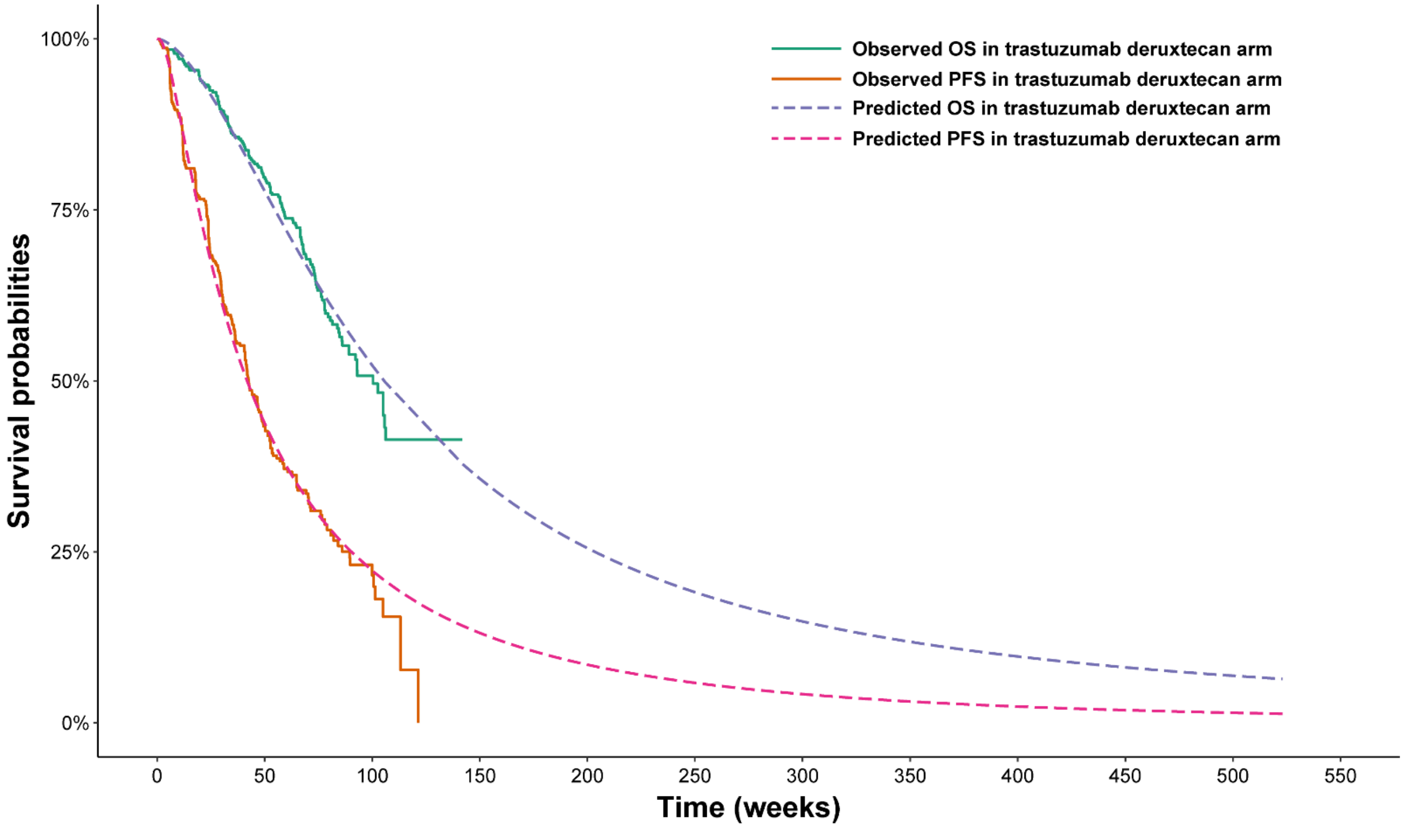

(B) Model-fitted versus original K-M curves for chemotherapy in all HER2-Low advanced BC patients, log-logistic and lognormal models were used to fit the OS and PFS K-M of chemotherapy.

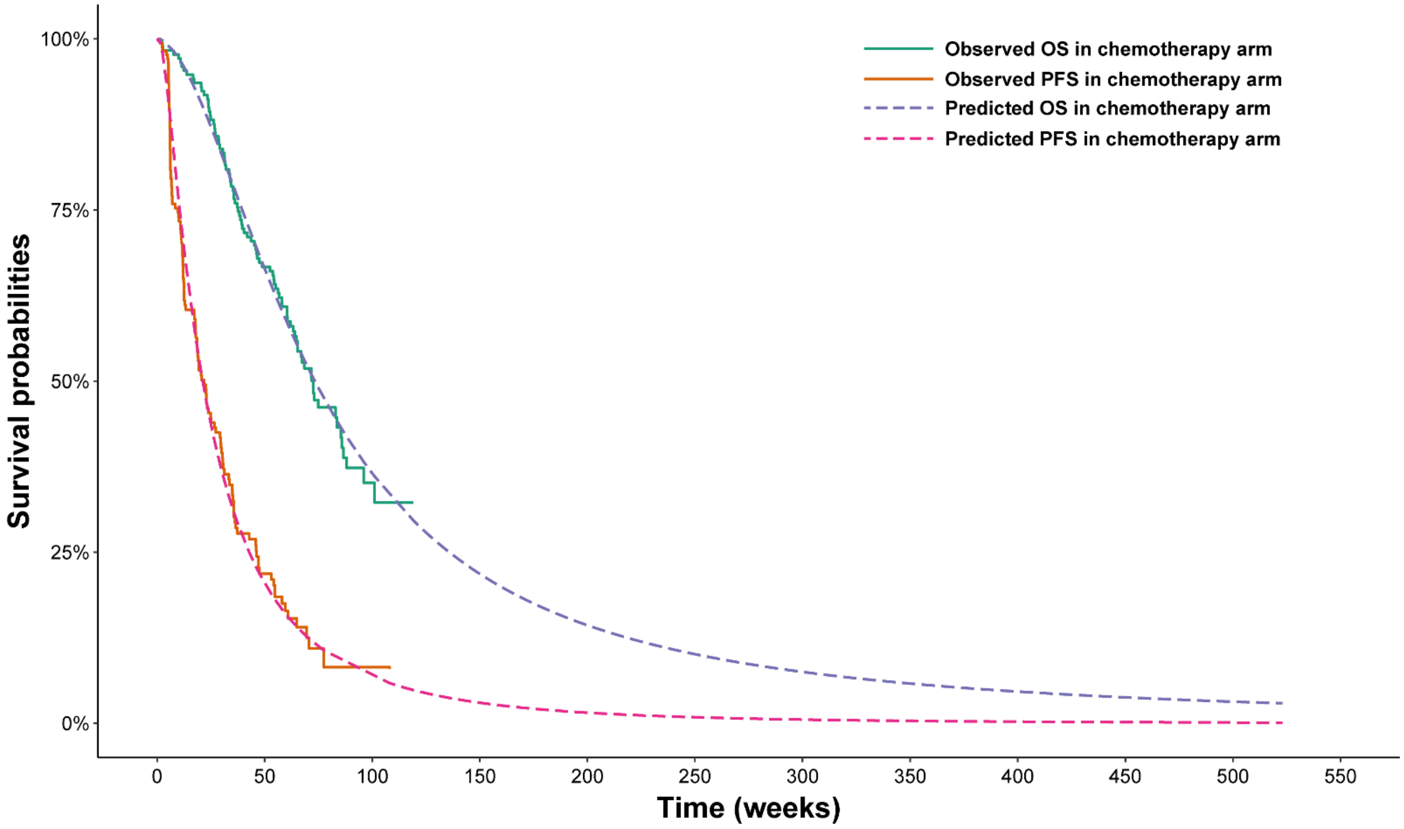

(C) Model-fitted versus original K-M curves for trastuzumab deruxtecan in HER2+ advanced BC patients, log-logistic and lognormal models were used to fit the OS and PFS K-M of trastuzumab deruxtecan.

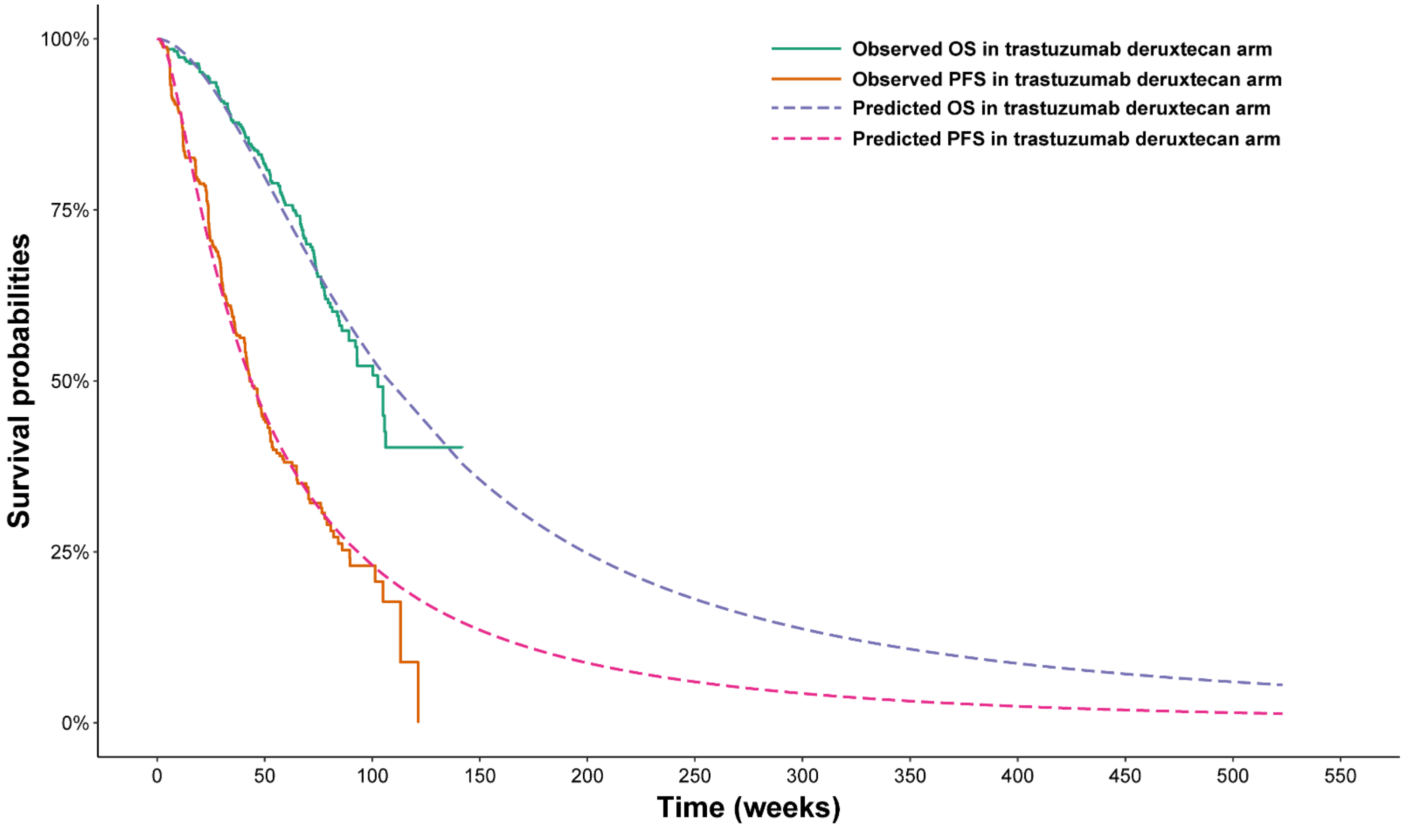

(D) Model-fitted versus original K-M curves for chemotherapy in HER2+ advanced BC patients, log-logistic and lognormal models were used to fit the OS and PFS K-M of chemotherapy.

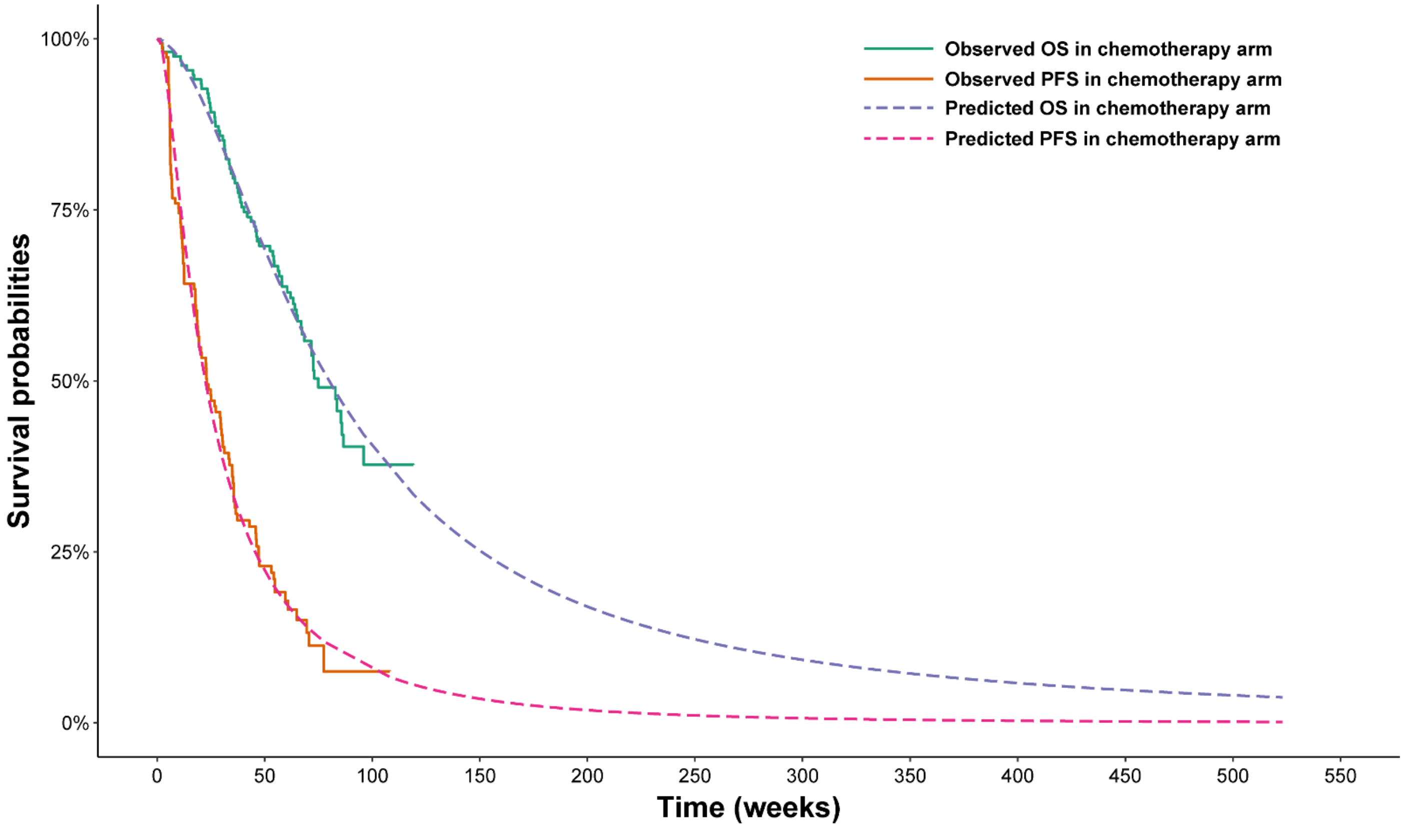

(E) Model-fitted versus original K-M curves for trastuzumab deruxtecan in HER2- advanced BC patients, log-logistic and lognormal models were used to fit the OS and PFS K-M of trastuzumab deruxtecan.

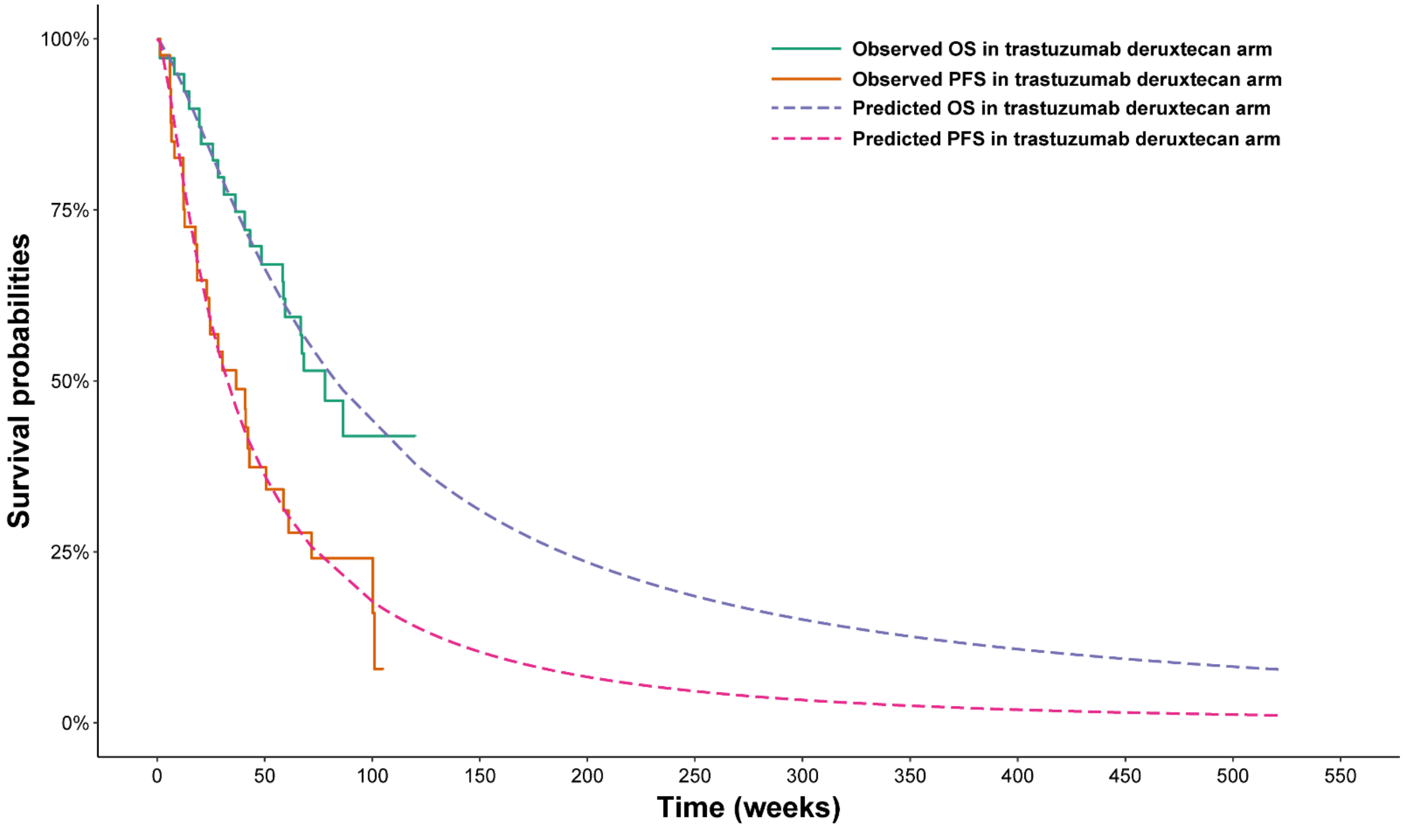

(F) Model-fitted versus original K-M curves for chemotherapy in HER2- advanced BC patients, log-lognormal model was used to fit the OS and PFS K-M of chemotherapy.

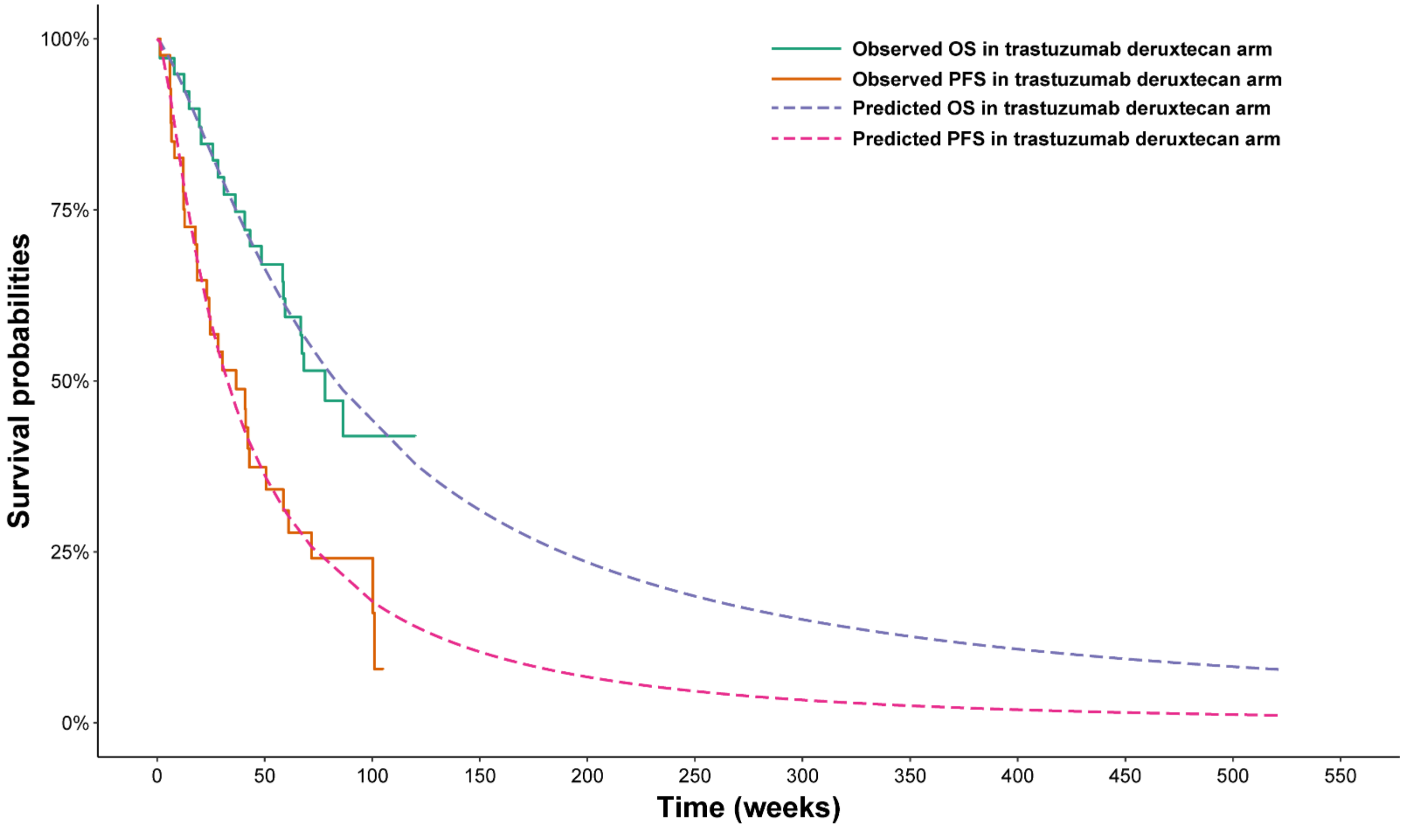

Supplement: S1 Fig — (PDF) [file pone.0290507.s001.pdf]
