# Supplementary material for: Cost-effectiveness of trastuzumab deruxtecan for previously treated HER2-low advanced breast cancer
Source: PLoS One. 2023 Aug 24;18(8):e0290507. doi: 10.1371/journal.pone.0290507 (PMC10449172; doi:10.1371/journal.pone.0290507)

S2 Fig. Tornado Diagram of 1-Way Sensitivity Analyses of Trastuzumab Deruxtecan Versus Chemotherapy. (A) All HER2-Low advanced BC patients, (B) HER2+ advanced BC patients, (C) HER2- advanced BC patients. BC, breast cancer; HER2, human epidermal growth factor receptor 2; HER2+, human epidermal growth factor receptor 2 positive; HER2-, human epidermal growth factor receptor 2 negative.

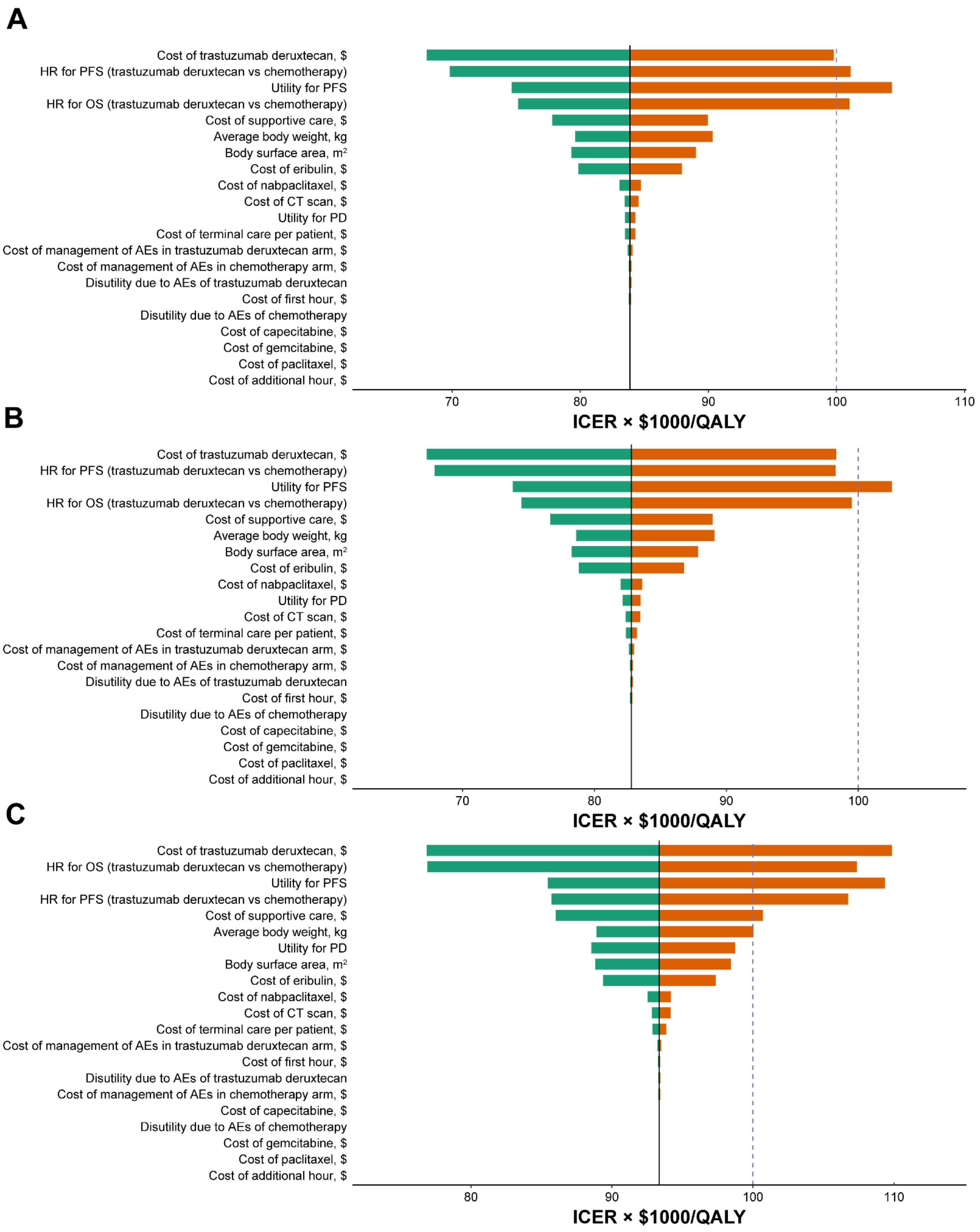

Supplement: S2 Fig — (PDF) [file pone.0290507.s002.pdf]
