# Supplementary material for: Cost-effectiveness of trastuzumab deruxtecan for previously treated HER2-low advanced breast cancer
Source: PLoS One. 2023 Aug 24;18(8):e0290507. doi: 10.1371/journal.pone.0290507 (PMC10449172; doi:10.1371/journal.pone.0290507)

S3 Fig. One-Way Sensitivity Analyses Result of ICER When Varying Cost of Trastuzumab Deruxtecan in Participants. Graphs represent the ICERs of trastuzumab deruxtecan compared with chemotherapy. BC, breast cancer; HER2, human epidermal growth factor receptor 2; HER2+, human epidermal growth factor receptor 2 positive; HER2-, human epidermal growth factor receptor 2 negative; ICER, Incremental cost-effectiveness ratio.

(A) All HER2-Low advanced BC patients.

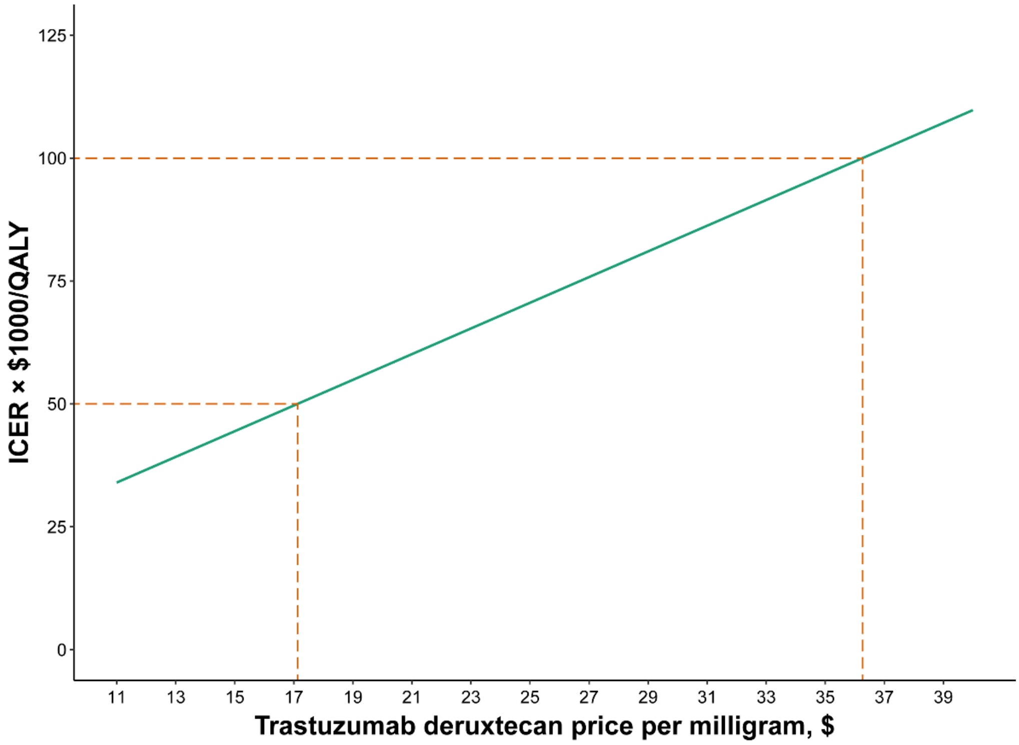

(B) HER2+ advanced BC patients.

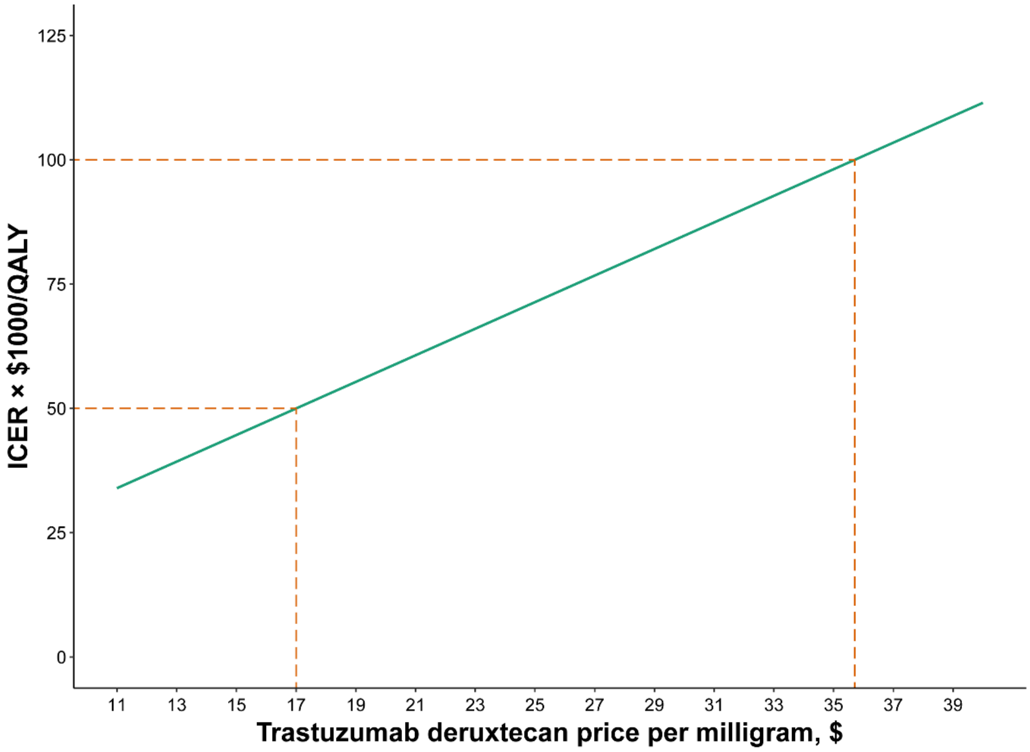

(C) HER2- advanced BC patients.

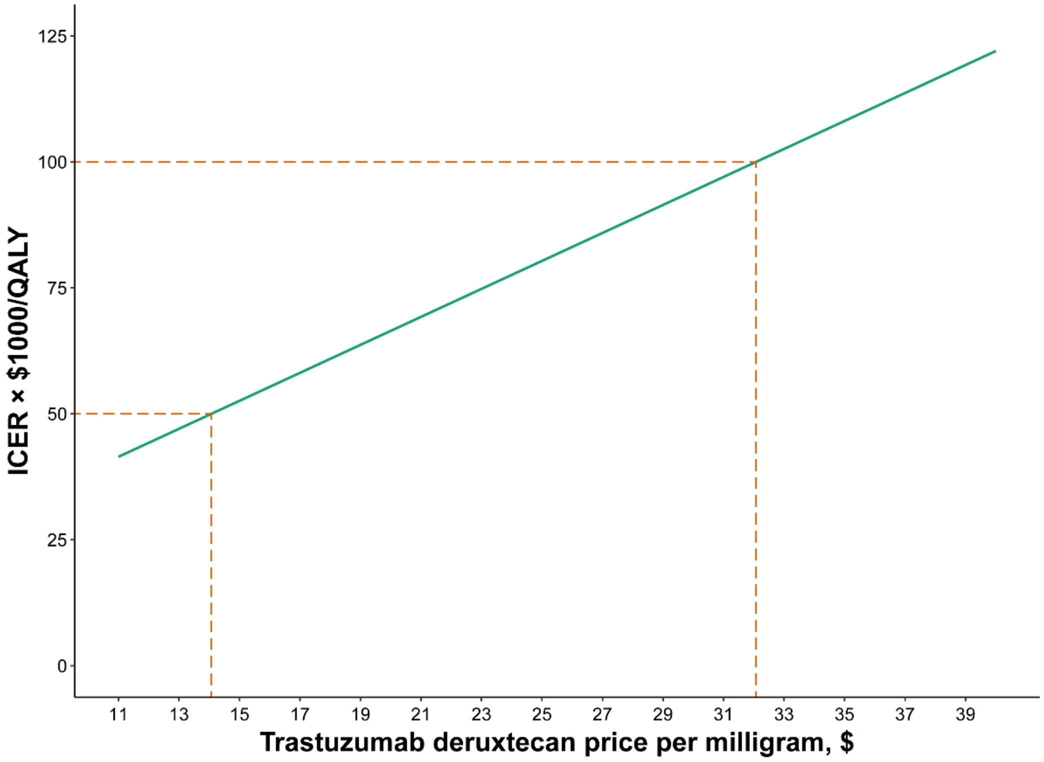

Supplement: S3 Fig — (PDF) [file pone.0290507.s003.pdf]
