# Supplementary material for: Cost-effectiveness of trastuzumab deruxtecan for previously treated HER2-low advanced breast cancer
Source: PLoS One. 2023 Aug 24;18(8):e0290507. doi: 10.1371/journal.pone.0290507 (PMC10449172; doi:10.1371/journal.pone.0290507)
Supplement: S1 Table — (PDF) [file pone.0290507.s004.pdf]

S1 Table. Estimated Parameters and AIC and BIC Values from Each Survival Model.

BC, breast cancer; HER2, human epidermal growth factor receptor 2; HER2+, human epidermal growth factor receptor 2 postive; HER2-, human epidermal growth factor receptor 2 negative.

(A) All HER2-Low advanced BC patients

Strategies	Distributions	Parameters	est	se	L95%	U95%	AIC	BIC
**Results of OS**								
**Trastuzumab Deruxtecan**	Exponential	rate	0.0060	0.0005	0.0051	0.0070	1849.15	1853.071
	WeibullPH	shape	1.4640	0.1063	1.2699	1.6879	1837.536	1845.379
		scale	0.0008	0.0004	0.0003	0.0020		
	Gamma	shape	1.6141	0.1579	1.3325	1.9551	1829.468	1837.311
		rate	0.0125	0.0019	0.0093	0.0168		
	Lognormal	meanlog	4.7468	0.0868	4.5768	4.9169	1849.773	1857.617
		sdlog	1.2020	0.0754	1.0629	1.3593		
	Gompertz	shape	0.0129	0.0027	0.0076	0.0181	1829.429	1837.272
		rate	0.0033	0.0005	0.0024	0.0046		
	Log-logistic	shape	1.6742	0.1206	1.4537	1.9281	**1828.881**	**1840.646**
		scale	105.5722	7.2335	92.3055	120.7456		
	Generalized gamma	mu	4.9020	0.0736	4.7577	5.0463	1831.065	1838.908
		sigma	0.5861	0.1324	0.3764	0.9125		
		Q	1.2675	0.3806	0.5216	2.0134		
**Chemotherapy**	Exponential	rate	0.0090	0.0009	0.0073	0.0110	1030.76	1033.975
	WeibullPH	shape	1.4661	0.1334	1.2266	1.7524	1017.6	1024.03
		scale	0.0012	0.0007	0.0004	0.0039		
	Gamma	shape	1.7197	0.2234	1.3331	2.2184	1017.166	1023.596
		rate	0.0189	0.0035	0.0131	0.0271		
	Lognormal	meanlog	4.3214	0.0978	4.1298	4.5130	1023.984	1030.413
		sdlog	1.0614	0.0844	0.9082	1.2404		
	Gompertz	shape	0.0118	0.0038	0.0044	0.0193	1023.451	1029.881
		rate	0.0056	0.0011	0.0038	0.0082		
	Log-logistic	shape	1.7826	0.1607	1.4940	2.1270	**1016.537**	**1022.966**
		scale	73.3379	6.0910	62.3207	86.3028		
	Generalized gamma	mu	4.5147	0.1060	4.3069	4.7224	1019.164	1028.809
		sigma	0.7570	0.1297	0.5411	1.0590		
		Q	0.7770	0.3169	0.1560	1.3981		
**Results of PFS**								
**Trastuzumab Deruxtecan**	Exponential	rate	0.0158	0.0010	0.0139	0.0179	2505.1	2509.022
	WeibullPH	shape	1.1856	0.0636	1.0672	1.3171	2497.768	2505.611
		scale	0.0075	0.0020	0.0045	0.0126		
	Gamma	shape	1.3170	0.1034	1.1292	1.5360	2495.56	2503.403
		rate	0.0223	0.0025	0.0179	0.0277		
	Lognormal	meanlog	3.7343	0.0652	3.6066	3.8620	**2493.641**	**2501.484**
		sdlog	1.1400	0.0544	1.0383	1.2517		
	Gompertz	shape	0.0046	0.0026	-0.0005	0.0097	2504.064	2511.907
		rate	0.0136	0.0015	0.0111	0.0168		
	Log-logistic	shape	1.5259	0.0820	1.3734	1.6953	2494.933	2502.776

		scale	42.5146	2.6413	37.6405	48.0197		
	Generalized gamma	mu	3.8903	0.1112	3.6724	4.1082	2494.052	2504.816
		sigma	1.0400	0.0847	0.8867	1.2200		
		Q	0.3706	0.2298	-0.0798	0.8210		
**Chemotherapy**	Exponential	rate	0.0297	0.0026	0.0250	0.0354	1149.05	1152.265
	WeibullPH	shape	1.1118	0.0757	0.9729	1.2706	1148.748	1155.177
		scale	0.0199	0.0057	0.0113	0.0350		
	Gamma	shape	1.2787	0.1361	1.0379	1.5753	1146.018	1152.448
		rate	0.0397	0.0058	0.0298	0.0528		
	Lognormal	meanlog	3.0558	0.0847	2.8897	3.2219	**1126.1**	**1134.53**
		sdlog	1.0395	0.0662	0.9176	1.1776		
	Gompertz	shape	-0.0023	0.0046	-0.0114	0.0069	1150.806	1157.236
		rate	0.0312	0.0041	0.0241	0.0404		
	Log-logistic	shape	1.6052	0.1137	1.3971	1.8442	1134.775	1141.204
		scale	20.9527	1.8407	17.6384	24.8896		
	Generalized gamma	mu	2.7914	0.1690	2.4601	3.1227	1126.946	1136.591
		sigma	1.0433	0.0679	0.9183	1.1853		
		Q	-0.5488	0.3012	-1.1392	0.0415		

(B) HER2+ advanced BC patients

Strategies	Distributions	Parameters	est	se	L95%	U95%	AIC	BIC
**Results of OS**								
**Trastuzumab Deruxtecan**	Exponential	rate	0.0057	0.0005	0.0048	0.0068	1581.613	1585.415
	WeibullPH	shape	1.5760	0.1237	1.3512	1.8381	1559.745	1567.349
		scale	0.0005	0.0003	0.0002	0.0014		
	Gamma	shape	1.7673	0.1884	1.4340	2.1779	1559.26	1568.865
		rate	0.0136	0.0022	0.0099	0.0188		
	Lognormal	meanlog	4.7748	0.0899	4.5986	4.9510	1577.206	1584.81
		sdlog	1.1403	0.0776	0.9979	1.3030		
	Gompertz	shape	0.0156	0.0029	0.0098	0.0213	1559.806	1567.41
		rate	0.0028	0.0005	0.0020	0.0039		
	Log-logistic	shape	1.7922	0.1401	1.5375	2.0890	**1559.315**	**1566.92**
		scale	107.6315	7.5334	93.8343	123.4574		
	Generalized gamma	mu	4.9029	0.0740	4.7579	5.0479	1569.841	1578.248
		sigma	0.5207	0.1340	0.3143	0.8624		
		Q	1.3428	0.4380	0.4843	2.2013		
**Chemotherapy**	Exponential	rate	0.0081	0.0009	0.0064	0.0102	851.1	854.194
	WeibullPH	shape	1.4523	0.1481	1.1893	1.7736	841.538	847.726
		scale	0.0012	0.0008	0.0003	0.0042		
	Gamma	shape	1.6640	0.2368	1.2590	2.1993	841.536	847.724
		rate	0.0168	0.0035	0.0111	0.0253		
	Lognormal	meanlog	4.4256	0.1144	4.2015	4.6498	848.378	854.565
		sdlog	1.1172	0.0990	0.9391	1.3290		
	Gompertz	shape	0.0122	0.0043	0.0038	0.0206	845.415	851.603
		rate	0.0050	0.0011	0.0032	0.0077		
	Log-logistic	shape	1.7295	0.1743	1.4194	2.1073	**841.225**	**847.413**
		scale	79.9403	7.5541	66.4248	96.2058		
		mu	4.6144	0.1130	4.3928	4.8359	843.445	852.726

	Generalized gamma	sigma	0.7310	0.1555	0.4818	1.1090		
		Q	0.8822	0.3699	0.1572	1.6072		
**Results of PFS**								
**Trastuzumab Deruxtecan**	Exponential	rate	0.0152	0.0010	0.0133	0.0174	2191.07	2194.873
	WeibullPH	shape	1.2142	0.0700	1.0844	1.3596	2182.675	2190.279
		scale	0.0064	0.0019	0.0036	0.0114		
	Gamma	shape	1.3619	0.1149	1.1543	1.6068	2180.591	2188.195
		rate	0.0225	0.0027	0.0178	0.0284		
	Lognormal	meanlog	3.7770	0.0686	3.6425	3.9115	**2179.548**	**2187.152**
		sdlog	1.1220	0.0575	1.0148	1.2406		
	Gompertz	shape	0.0054	0.0028	0.0000	0.0109	2189.349	2196.953
		rate	0.0128	0.0015	0.0102	0.0160		
	Log-logistic	shape	1.5574	0.0901	1.3905	1.7443	2179.994	2187.599
		scale	44.3117	2.8762	39.0182	50.3233		
	Generalized gamma	mu	3.9351	0.1156	3.7084	4.1617	2179.641	2190.547
		sigma	1.0158	0.0915	0.8513	1.2121		
		Q	0.3879	0.2478	-0.0977	0.8736		
**Chemotherapy**	Exponential	rate	0.0283	0.0027	0.0235	0.0341	1015.059	1018.153
	WeibullPH	shape	1.1392	0.0837	0.9864	1.3157	1014.099	1020.287
		scale	0.0172	0.0055	0.0092	0.0321		
	Gamma	shape	1.3017	0.1480	1.0418	1.6266	1012.002	1018.19
		rate	0.0387	0.0060	0.0285	0.0524		
	Lognormal	meanlog	3.1119	0.0914	2.9327	3.2911	**1001.234**	**1007.421**
		sdlog	1.0497	0.0713	0.9188	1.1992		
	Gompertz	shape	0.0010	0.0049	-0.0085	0.0105	1017.018	1023.206
		rate	0.0277	0.0040	0.0209	0.0368		
	Log-logistic	shape	1.5937	0.1207	1.3738	1.8489	1006.917	1013.104
		scale	22.5424	2.1254	18.7389	27.1178		
	Generalized gamma	mu	2.9797	0.1887	2.6098	3.3496	1002.581	1011.862
		sigma	1.0676	0.0737	0.9325	1.2222		
		Q	-0.2760	0.3400	-0.9424	0.3904		

(C) HER2- advanced BC patients

**Strategies**	**Distributions**	**Parameters**	**est**	**se**	**L95%**	**U95%**	**AIC**	**BIC**
**Results of OS**								
**Trastuzumab Deruxtecan**	Exponential	rate	0.0082	0.0018	0.0053	0.0126	245.788	247.477
	WeibullPH	shape	1.1184	0.2197	0.7609	1.6437	247.478	250.856
		scale	0.0049	0.0048	0.0007	0.0334		
	Gamma	shape	1.1742	0.3095	0.7005	1.9683	247.43	250.807
		rate	0.0103	0.0044	0.0045	0.0237		
	Lognormal	meanlog	4.4562	0.2662	3.9345	4.9779	248.595	251.973
		sdlog	1.3994	0.2361	1.0054	1.9480		
	Gompertz	shape	0.0014	0.0076	-0.0135	0.0163	247.753	251.131
		rate	0.0077	0.0030	0.0036	0.0164		
	Log-logistic	shape	1.3437	0.2581	0.9222	1.9579	**247.203**	**250.581**
		scale	83.0066	18.7408	53.3252	129.2089		
	Generalized gamma	mu	4.6960	0.2907	4.1263	5.2657	249.385	254.452
		sigma	1.0017	0.3962	0.4614	2.1746		

		Q	0.7673	0.7207	-0.6452	2.1799		
**Chemotherapy**	Exponential	rate	0.0182	0.0047	0.0110	0.0301	152.249	153.14
	WeibullPH	shape	1.5091	0.3163	1.0007	2.2758	151.535	153.316
		scale	0.0022	0.0030	0.0002	0.0314		
	Gamma	shape	1.9220	0.6418	0.9989	3.6983	151.98	152.861
		rate	0.0367	0.0146	0.0168	0.0801		
	Lognormal	meanlog	3.6921	0.2064	3.2876	4.0966	**151.009**	**152.789**
		sdlog	0.8377	0.1557	0.5819	1.2059		
	Gompertz	shape	0.0161	0.0092	-0.0019	0.0342	151.347	153.128
		rate	0.0097	0.0047	0.0037	0.0252		
	Log-logistic	shape	2.0303	0.4323	1.3376	3.0818	152.155	153.936
		scale	41.5880	8.7215	27.5715	62.7299		
	Generalized gamma	mu	3.9797	0.4434	3.1108	4.8487	152.978	155.649
		sigma	0.7082	0.2789	0.3273	1.5324		
		Q	0.7823	1.1726	-1.5158	3.0805		
**Results of PFS**								
**Trastuzumab Deruxtecan**	Exponential	rate	0.0192	0.0035	0.0134	0.0274	299.26	300.949
	WeibullPH	shape	1.0495	0.1585	0.7806	1.4111	301.16	304.537
		scale	0.0157	0.0104	0.0043	0.0571		
	Gamma	shape	1.1162	0.2495	0.7202	1.7299	301.024	304.402
		rate	0.0219	0.0069	0.0118	0.0406		
	Lognormal	meanlog	3.4850	0.2016	3.0899	3.8801	**299.852**	**303.23**
		sdlog	1.2110	0.1632	0.9299	1.5770		
	Gompertz	shape	-0.0005	0.0074	-0.0150	0.0140	301.256	304.634
		rate	0.0194	0.0057	0.0110	0.0345		
	Log-logistic	shape	1.4069	0.2122	1.0468	1.8908	300.484	303.862
		scale	33.0842	6.6374	22.3280	49.0219		
	Generalized gamma	mu	3.5864	0.3702	2.8609	4.3119	301.751	306.817
		sigma	1.1696	0.2129	0.8186	1.6709		
		Q	0.2018	0.6346	-1.0419	1.4455		
**Chemotherapy**	Exponential	rate	0.0413	0.0110	0.0245	0.0697	119.237	120.127
	WeibullPH	shape	1.0670	0.2054	0.7316	1.5561	121.128	122.909
		scale	0.0329	0.0247	0.0076	0.1432		
	Gamma	shape	1.3343	0.4394	0.6998	2.5444	120.523	122.304
		rate	0.0572	0.0248	0.0245	0.1336		
	Lognormal	meanlog	2.7318	0.2220	2.2968	3.1668	**115.562**	**117.343**
		sdlog	0.8899	0.1714	0.6101	1.2982		
	Gompertz	shape	-0.0132	0.0154	-0.0433	0.0170	120.395	122.176
		rate	0.0523	0.0188	0.0259	0.1056		
	Log-logistic	shape	1.9466	0.4282	1.2648	2.9959	115.657	117.438
		scale	14.0534	3.1126	9.1045	21.6923		
	Generalized gamma	mu	1.7320	NA	NA	NA	116.284	118.956
		sigma	0.0638	NA	NA	NA		
		Q	-17.3737	NA	NA	NA		
