# Supplementary material for: Cost-effectiveness of trastuzumab deruxtecan for previously treated HER2-low advanced breast cancer
Source: PLoS One. 2023 Aug 24;18(8):e0290507. doi: 10.1371/journal.pone.0290507 (PMC10449172; doi:10.1371/journal.pone.0290507)
Supplement: S2 Table — (PDF) [file pone.0290507.s005.pdf]

S2 Table. Associated Costs and Disutility of Grade ≥ 3 Treatment-Related Adverse Events.

Adverse Event[a]	No. of patients (%)[b]	Costs in 2021 USDc	Reference	Disutility	Reference
**Trastuzumab Deruxtecan**					
Febrile neutropenia	51 (13.7)	9,649	[1]	0.030	[1]
Anemia	30 (8.1)	15,196	[2]	0.073	[2]
Thrombocytopenia	19 (5.1)	20,972	[3]	0.066	[3]
Leukopenia	24 (6.5)	4,934	[1]	0.003	[1]
Nausea/vomiting	22 (5.9)	8,019	[3]	0.088	[3]
Diarrhea	4 (1.1)	7,456	[3]	0.103	[3]
Fatigue	28 (7.5)	1,172	[3]	0.099	[3]
**Total**[c]		4,586		0.0273	
**Chemotherapy**					
Febrile neutropenia	70 (40.7)	9,649	[1]	0.030	[1]
Anemia	8 (4.7)	15,196	[2]	0.073	[2]
Thrombocytopenia	1 (0.6)	20,972	[3]	0.066	[3]
Leukopenia	33 (19.2)	4,934	[1]	0.003	[1]
Nausea/vomiting	0 (0)	8,019	[3]	0.088	[3]
Diarrhea	3 (1.7)	7,456	[3]	0.103	[3]
Fatigue	8 (4.7)	1,172	[3]	0.099	[3]
**Total**[c]		5,896		0.0230	

[a]Our analysis only included and evaluated grade ≥ 3 treatment-related adverse events.

[b]Number within treatment arm: trastuzumab deruxtecan (N = 371), chemotherapy (N = 172).

[c]Calculated as an average cost of toxicity using the weighted frequency of occurrence. This value was used in the base-case model.

References

1. Jeong, E., Wang, C., Wilson, L., and Zhong, L. (2021). Cost-Effectiveness of Adding Ribociclib to Endocrine Therapy for Patients With HR-Positive, HER2-Negative Advanced Breast Cancer Among Premenopausal or Perimenopausal Women. *Front Oncol* 11**,** 658054. doi: 10.3389/fonc.2021.658054.

2. Wang, H., Wang, Y., Gong, R., Geng, Y., and Li, L. (2021). Cost-effectiveness of pertuzumab and trastuzumab as a first-line treatment of HER2-positive metastatic breast cancer in China. *Ann Palliat Med* 10(11)**,** 11382-11393. doi: 10.21037/apm-21-2412.

3. Diaby, V., Adunlin, G., Ali, A.A., Zeichner, S.B., de Lima Lopes, G., Kohn, C.G., et al. (2016). Cost-effectiveness analysis of 1st through 3rd line sequential targeted therapy in HER2-positive metastatic breast cancer in the United States. *Breast Cancer Res Treat* 160(1)**,** 187-196. doi: 10.1007/s10549-016-3978-6.
